# Emotion Perception in Members of Norwegian Mensa

**DOI:** 10.3389/fpsyg.2019.00027

**Published:** 2019-01-23

**Authors:** Jens Egeland

**Affiliations:** Vestfold Hospital Trust, Division of Mental Health and Addiction, Department of Psychology, University of Oslo, Oslo, Norway

**Keywords:** superior intelligence, emotion recognition, EmoBio, Mensa, positive manifold

## Abstract

Are people with superior intelligence also superior in interpreting the emotions of others? Some studies find that an underlying g-factor links all mental processes leading to an expectation of a positive answer to the question, while other studies find that there is a cost to giftedness. No previous study have tested social cognition among highly gifted, or the Mensa society specifically. The study measures emotion recognition in 63 members of the Norwegian Mensa and 101 community controls. The Mensa group had a higher total score on the EmoBio test and was specifically better at differentiating the anger emotion, otherwise hypothesized to be mediated by subcortical processes. There was no difference in heterogeneity between the groups, contrary to the expectation of an autistic subgroup in Mensa. The study indicate that the positive manifold extends also to social cognition, and runs counter to the concept of a cost to giftedness.

## Introduction

Are people of superior intelligence also superior in social cognition or specifically in interpreting the emotions of others? The concept of “positive manifold” suggests that would be the case (Chabris, 2006). Although it may not be the causative agent, an underlying g-factor is systematically identified statistically, and contributes to better performance in all kinds of mental processes (Chabris, 2006). Contrary to this, the newly presented “hyper brain /hyper body”-theory suggests that superior intelligence, i.e., intellectual over-excitability is related to general psychological and indeed also physiological over-excitability causing higher rate of disorders such as autism spectrum disorders and ADHD in people with superior intelligence (Karpinsky et al., 2018). Based on a survey to members of the American Mensa they found a higher prevalence of these neurodevelopmental disorders, but also a higher prevalence of anxiety and mood disorders. As the study was based on self-report, it is a risk of over-reporting, but it might also be that people with superior intelligence make more use of health services and thus receive diagnoses more often than the population at large. However, also the newly presented “high intelligence imbalance hypothesis” (Crespi, 2016) claim that there is a cost to high intelligence. The model is based on the presumed overlap between the phenotypes of high intelligence and the phenotypes of autism. The claim is based on a review of associations between technical interest, gender differences in types of intelligence and higher male prevalence of autism. The study argues for the plausibility for the hypothesis rather than being a direct test of it. However, testing the association between high intelligence and social cognition by self-ratings, Machu and Cervinkova (2014) found a negative relation to some aspects of social cognition. They differentiated between social information processing, social skills and social awareness, and found that intellectually superior children were inferior to average children in the latter two aspects of social cognition. There were no difference with regard to social information processing, which is the topic of the present study: We are interested in whether high intelligence, which by definition is related to superior information processing of non-social stimuli, also leads to superior processing of social stimuli. Studies of clinical samples show a correlation between intelligence and social cognition (Egeland et al., 2017), but one hypothesis is that this is due to a threshold effect: some level of general cognitive resources is necessary for efficient social information processing. Above this threshold, there will no correlation (Fanning et al., 2012). If this is the case, people with superior intelligence should not be better than the population at large in processing social information.

The Mensa society is an organization for people with an IQ of at least 130, i.e., belonging to the most intelligent two percent of the population. While both the Karpinsky and Machu –studies relied on subjective information, i.e., information given by the subject themselves, no previous study have tested social skills objectively among subjects with superior intelligence. In the present study, we apply an emotion recognition test to members of the Norwegian Mensa and compare the results to that of community controls.

When testing whether the positive manifold extends to emotion recognition, it may be necessary to distinguish between emotions. Previous studies of emotion recognition from faces show that anger and fear is least accurately identified (Williams et al., 2009). Imaging studies show that anger is related to anterior cingulate and orbitofrontal cerebral activation (Williams et al., 2005) while sadness and happiness are related to a more distributed cerebral activation pattern. A hypothesis would be that negative emotions like anger and fear is more related to automatic subcortical processing, while positive emotions and sadness would be more related to cognitive processing.

Three questions are asked in the present study:

(1) Is there a group difference between the samples? Better performance among the Mensa members would support the idea that the positive manifold extends also to perception of emotional stimuli. On the other hand, impaired performance could be taken in support of the over-excitability model by Karpinsky et al. (2018).
(2) Is the group difference related to more accurate interpretation of specific emotions such as positive and neutral emotions on the one hand and anger and fear on the other hand? Having less deeply rooted survival value, the positive emotions would be expected to be more cognitively assessed, while anger and fear are processed more automatically. Types of errors made could also give information about possible emotional bias. Would any of the groups have a more conservative interpretative style, answering no emotion (neutral) where is in fact an emotion? Would any of the groups have a more negative or positive bias, interpreting positive film clips as negative or vice versa?
(3) The hyper brain and hyper body model by Karpinsky et al. (2018) suggests that superior intelligence is related to increased risk of autism spectrum disorders. Also, the imbalance model of Crespi (2016) describes a risk of imbalanced development of intelligence, resulting in more prevalent autism-related impairments like emotion recognition in superior intelligence. However, both the “positive manifold” phenomenon *and* imbalanced development could operate simultaneously. In that case, the Mensa group will perform average, but the heterogeneity in scores would be expected to be larger than among community controls. Thus, heterogeneity is examined: larger variance in performance in the superior IQ group would be taken in support of Karpinsky et al. (2018). Larger variance in the unselected community controls would be taken as an indication of the positive manifold hypotheses, as these subjects are expected to vary more also in intelligence.

All the above sets of analyses could be also yield insignificant findings. In that case, one would think that measuring emotional perception is altogether not related to intelligence. Machu and Cervinkova (2014) differentiated between social information processing, social abilities and social awareness. The negative correlations between superior intelligence and social ability were related to the latter two aspects. Regarding information processing they found a trend level (*p* = 0.067) and concluded negatively. Emotion perception tested in the present study, would be considered as a test of social information processing. We do not know how people who score high or low, actually apply their ability, i.e., their social skills.

## Materials and Methods

### Participants

Sixty-three members of Mensa attending a national meeting of the association in 2017, participated in the study. They gave information as to education, age and gender and there were no exclusion criteria. The comparison group was drawn from two settings: Senior high school students above the age of 18 participating in a psychology course and employees in a governmental agency. The school, the agency and the Mensa organization approved the inquiry to collect data. The consent to participate was written and informed from participants. None of the participants were patients, and prior to the implementation of GDPR, an ethics or data-inspectorate approval of the protocol was not required by the researcher’s institution nor by the Norwegian Data Inspectorate for collecting anonymous data.

Information about gender composition, age and education is presented in Table 1. The comparison group was significantly younger and had more female participants. Thus, age and gender was controlled for in the analyses.

**Table 1:** Demographic information.

	Mensa	Community sample
Males/females	39/24	67/24^∗^
Age	38.0 (8.9)	29.0 (15.6)^∗^
Education		
% high school	3	63
% vocational education	3	22
% bachelor degree	22	14
% master degree	42	18


*^∗^p < 0.001*.

### Instruments

In the Emotion in Biological Motion test (EmoBio: Heberlein et al., 2004) the subject is presented 22 short film clips of point-light display walkers pretending to express four emotions or being emotion neutral. The clips were projected to a wall screen, while the participants sequentially indicated on a sheet of paper which emotion was displayed. The emotions were angry, happy, sad, fearful, or neutral/no emotion. We used the same proportional scoring method as previous studies (Heberlein et al., 2004; Couture et al., 2010; Vaskinn et al., 2016). Some of the clips are easy to interpret, others are more ambiguous. A response is given credit based on the proportion of healthy control participants giving that response. The EmoBio test was chosen for this study because of newly developed Norwegian standardization data (Vaskinn et al., 2016). The standardization group consisted of 101 healthy control participants, randomly selected from national statistical records. Responding as the majority of the standardization group would give a score of 1. Other responses could be given a score ranging from zero to 0.43 depending on the frequency of that response in the standardization sample. The EmoBio test is increasingly applied in social neuroscience, as a measure of social cognition mediating between general cognition and outcome measures of community functioning (Olbert et al., 2013).

### Statistical Analyses

The EmoBio scores of the two groups were compared with Analysis of Variance and thereafter with Analysis of Covariance, controlling for age and gender effects. Then the separate scores for the four emotions as well as the neutral condition were entered as within-subjects variable and group as between subject variable in a Repeated Measures Analysis of Variance. This analysis was also repeated with age and gender as covariates. Although high education could be considered associated with intelligence, we also added education as a third covariate to reduce the impact of potential confounders. Which emotions that contribute to a possible significant group difference were analyzed with a series of Analyses of Variance (ANOVA) and ANCOVAs covarying gender and age. Alpha level was set to 0.0125 correcting for multiple comparisons. The effect of potential significant covariates were explored further by correlations between the covariates and total EmoBio score as well as for possible significant single emotions. The correlations were run both for the total sample and for the each group.

Errors were classified into five types: (1) Neutral (no emotion) response when in fact the majority of the standardization sample had seen an emotion; (2) emotion where the majority answered with an emotion; changing the quality of the emotion from (3) negative to positive or (4) from positive to negative; (5) classification errors within negative emotions. The error types were analyzed with Repeated Measures ANOVA and then with Repeated Measures MANCOVA with age and gender as covariates. To test the hypothesis of unequal heterogeneity each person’s deviation from his or her respective group mean was computed irrespective of whether the deviation was negative or positive. The mean deviation score in the two groups was then compared with ANOVA.

## Results

Table 2 shows the average score pr. item for the complete EmoBio test for both groups as well as the average score pr. item related to specific emotions. The table also shows the *p*-values and partial η^2^-values for the main effect of group when controlling for age and gender.

**Table 2:** Group comparison mean EmoBio score pr. item and mean scores for each emotion.

	Group comparison					Group comparison controlling for age and gender	
	Mensa *N* = 63	Community sample *N* = 101	*F*	*P*	Partial η^2^	*p*	Partial η^2^
EmoBio total^1,2^	0.857 (0.083)	0.816 (105)	5.831	0.017	0.035	<0.001	0.094
Sadness^1,3^	0.865 (0.150)	0.856 (0.169)	0.103	n.s.	0.001	n.s.	0.005
Happiness^1,3^	0.892 (0.120)	0.858 (0.170)	1.902	n.s.	0.012	n.s.	0.019
Fear^1,3^	0.814 (0.207)	0.840 (0.221)	0.529	n.s.	0.003	n.s.	0.002
Anger^1,3^	0.797 (0.169)	0.702 (0.193)	10.287	0.002	0.060	<0.001	0.188
Neutral^1,3^	0.892 (0.194)	0.837 (0.174)	3.522	n.s.	0.021	n.s.	0.035


*^1^Mean score pr. Item (1 = all correct), ^2^ANOVA/ANCOVA, and ^3^Repeated Measures ANOVA/ANCOVA*.

There was a significant group difference in total EmoBio score (1, 163) 5.831, *p* = 0.017, partial η^2^ = 0.035. The Mensa group scored above the community controls. Entering the five emotions in a Repeated Measures ANOVA also showed a significant main effect of group [*F* = (1, 161) 4.448, *p* = 0.036, partial η^2^ = 0.027] and a significant within subjects x group interaction (Greenhouse-Geisser: *F* = 2.848, *p* = 0.027, partial η^2^ = 0.017). The two groups differed only with regard to Anger [*F* = (1, 164) 10.923, *p* = 0.001, partial η^2^ = 0.063].

Controlling for age and gender showed that age [*F* = (5, 155) 9.390, *p* < 0.001, partial η^2^ = 0.232] was related to performance while gender was not [*F* = (5,155) 1.944, *p* = 0.090, partial η^2^ = 0.059]. When controlling for age and gender, the main effect of group increased [*F* = (5, 155) 8.509, *p* < 0.001, partial η^2^ = 0.215]. Repeated Measures ANOVA showed an increased effect of group [*F* = (1, 161) 12.767, *p* < 0.001, partial η^2^ = 0.074] and a significant within subjects interaction (Greenhouse-Geisser: *F* = 5.925, p < 0.001, partial η^2^ = 0.036). Adding education as a covariate, the between group effect remained significant [*F* = (1, 172) 8.024, *p* = 0.005, partial η^2^ = 0.045]. In this sample, education was not a significantly related to performance. With education as a third covariate, the within subjects interaction became insignificant. When covarying age and gender, Anger remained significant and there was a tendency also for a group difference in neutral stimuli (*p* = 0.018).

Younger age was associated with better performance for the complete EmoBio test (*r*^2^ = -0.227, *p* = 0.004) and the Anger-score (*r*^2^ = -0.392, *p* < 0.001). Correlations within each group showed that both scores remained associated to age in the community sample (EmoBio total score: *r*^2^ = -0.371, *p* < 0.001, Anger: *r*^2^ = -0.597, *p* < 0.001). In the Mensa sample only Anger correlated significantly with age (*r*^2^ = -0.251, *p* = 0.047).

Table 3 shows the types of errors made. Both before and after controlling for age and gender, there were significant between subjects effects [*F* = (1, 175) 7.800, *p* = 0.005, partial η^2^ = 0.043; covarying age and gender: *F* = (1, 173) 12.459, *p* = 0.001, partial η^2^ = 0.067]. Age and gender were not significantly associated to error types. There were no significant group-error type interaction. Both groups differed with regard to number of type 5 errors, i.e., errors within the negative spectrum. Controlling also for differences in total numbers of errors, the type 5 group difference was reduced to a tendency [*F* = (4,163) 3.860, *p* = 0.051, partial η^2^ = 0.024].

**Table 3:** Types of errors made.

	ANOVA					ANCOVA controlling for age and gender	
	Mensa *N* = 63	Community sample *N* = 101	*F*	*P*	Partial η^2^	*p*	Partial η^2^
Neutral instead of emotion^1^	1.22 (1.36)	1.32 (1.59)	0.156	n.s.	0.001	n.s.	0.006
Emotion instead of neutral	0.46 (0.82)	0.64 (0.72)	2.402	n.s.	0.013	n.s.	0.018
Positive instead of negative	0.73 (0.84)	0.94 (0.97)	2.045	n.s.	0.011	n.s.	0.023
Negative instead of positive	0.38 (0.52)	0.43 (0.63)	0.232	n.s.	0.001	n.s.	0.000
Errors within negative emotions	1.21 (1.08)	1.69 (1.19)	7.040	0.009	0.038	0.007	0.041


*^1^All scores are average number of errors of each type*.

Regarding the question of unequal heterogeneity, the Mensa group had a mean of 1.46 score difference (s.d. 1.08) between the mean total score and individual scores, while the equivalent figure was 1.84 in the comparison group (s.d. 1.37). This difference was not significant (*p* = 0.068).

## Discussion

In the present study we compare members of the Norwegian Mensa to a community control group on a test of emotion recognition that has previously shown impaired performance among subjects with high functioning autism (Couture et al., 2010) and that subjects with schizophrenia are impaired beyond the effect of reduced IQ (Egeland et al., 2017).

The idea that there is a cost to giftedness is old. The notion of the “mad genius” was particularly popular in the late Victorian era (Stiles, 2009). However, one could claim that there is a continuity between that notion and the models presented by Crespi (2016) and Karpinsky et al. (2018). As an exponent of the original “mad genius”- concept, John Ferguson Nisbet claimed in his book “the Insanity of Genius” from 1891 that all kinds of deviations from the normal, be it superiority or inferiority, was necessarily pathological and represented a “nerve disorder” (Stiles, 2009). The opposite view in the following “mad genius controversy” was held by Galton and Terman. Transcending the anecdotal evidence dominating the debate to that time, Terman studied gifted students empirically and concluded that they were not only intellectually gifted, but were generally also socially well adapted (Terman, 1922). This view ran contrary to the view by Hollingworth (1931) that gifted children typically had problems with social adjustment. She recommended special classes for highly intelligent children. Nevertheless, neither she found that highly intelligent children were impaired in social cognition, but claimed instead that they were not understood by the “bullies” of less intelligence. A recent study by Baudson (2016) found that the notion of the “mad genius” is alive and well in the sense that what she characterize as “the envious stereotype” that gifted persons are competent but emotionally cold is held by a majority of Germans. She warns that such a view can contribute to out-grouping of gifted persons. The views of Crespi (2016) and Karpinsky et al. (2018) that there are costs to being intellectually gifted could be considered a modern variant of the “mad genius” hypothesis, and should be assessed critically so as not to unjustly support popular prejudice.

The present study cannot claim to be a critical test of whether there are costs to being intellectually gifted in general, but it is at least a test of *one* such aspect, – emotion recognition. On the one hand, emotion recognition is impaired in subjects with high functioning autism (Couture et al., 2010). If a cost of giftedness is higher prevalence of autistic symptoms, we should expect that intellectually gifted are impaired on the EmoBio test as well. On the other hand, -although emotion recognition represent a separate and distinct ability from intelligence among subjects with schizophrenia (Egeland et al., 2017), it still correlates somewhat with intelligence. In this study we find that the Mensa members perform better than the community comparison sample, indicating that the “positive manifold” extends to this aspect of social cognition, rather than being unrelated or negatively related to intelligence.

Splitting up the total score into separate emotions, showed that only anger differed between the two groups. However, for the remaining emotions the directions of results were also in favor of the Mensa group. Thus, no interaction between giftedness and survival value of emotions was found, as one could suspect if anger and fear were processed in more robust subcortical pathways, while positive emotions were processed in cortical pathways expected to correlate higher with intelligence. Checking the findings of other studies of emotion recognition, however, shows that anger seems to differentiate more between groups simply because anger seems to be the most difficult emotion to process. Both subjects with Down syndrome (Cebula et al., 2017) and people suffering from Traumatic Brain Injury (Rosenberg et al., 2014) are impaired in emotion recognition, but more so in anger compared to other emotions. Both these clinical groups are impaired also in non-social cognition. However, as in the present study, also the control groups of the cited studies have a lower score on anger than other emotions. The discriminating power of a variable is largest when accuracy level approaches 50% (Miller et al., 1995). Ideally, all emotions should be matched for accuracy level in order to reliably identify group differences with regard to one of them (Chapman and Chapman, 2001). Anger had the lowest correct score both in this study and in the healthy control group of Vaskinn et al. (2016). Thus, we conservatively interpret the group difference in Anger alone (and not fear) as a result of somewhat better discriminating power of this emotion, making this emotion statistically significant and merely a tendency for better performance of Mensa-members for the other emotions.

Interpreting emotions as neutral (error type 1) and interpreting a negative emotion as another negative emotion were the two most frequent error types in both groups. There were no tendency for the Mensa group to interpret more conservatively, but there were a group difference in type 5 errors, – i.e., not being able to differentiate between negative emotions. When controlling also for the differences in total errors, the Mensa superiority in discriminating among negative emotions, was reduced to a tendency. More refined research controlling for baserate differences in errors of detecting emotions could reveal differences, but presently we also here must conclude conservatively that there does not seem to be robust group related differences in emotional bias.

In the introduction we presented the possibility that both the positive manifold phenomenon and the cost to giftedness- models could have some merit simultaneously. Overall, intelligence may favor emotion recognition, but at the same time, a higher percentage of highly intelligent subjects may be specifically impaired because of a higher incidence of autism spectrum disorders. We tested this by analyzing heterogeneity within the two groups. Numerically, the deviation was smaller within the Mensa group, but the difference was not significant. Thus, we find no evidence of larger heterogeneity as would be expected if high intelligence is a risk factor for autism spectrum disorders.

There are some limitations to the study: We do not have the actual intelligence score of the participants. On the other hand, this is a group comparison design, and it seems reasonable to expect that the Mensa members are actually of higher intelligence than the community sample, due to the intelligence test needed to join the society. Under other conditions, education could have served as a proxy of intelligence, but the young age of many of the participants prohibits this, as their low education was due to age more than capacity. Including participants from a government directorate insures us that the group difference is not due to low intellectual capacity of the comparison group. The scores of the community group is similar to that of Vaskinn et al. (2016).

The present study differs from the study by Karpinsky et al. (2018) in design and in overall findings. However, we also want to emphasize that the samples differs with regard to nationality, i.e., the present study compares *Norwegian* Mensa members to *Norwegian* control subjects. Little is known about cultural differences in emotion perception, but one cannot rule out that such differences exist, limiting the generalization of the findings.

Summing up the study, it indicates that the positive manifold of IQ extends into social cognition, and runs contrary to what would be expected from the Karpinsky et al. (2018) study, expecting a social cognitive cost of being intellectually gifted.

## Author Contributions

JE performed the data collection, analyses, and wrote the manuscript.

## Conflict of Interest Statement

The author declares that the research was conducted in the absence of any commercial or financial relationships that could be construed as a potential conflict of interest.
